# Real-World 30-Day Mortality After the Last Dose of Immune Checkpoint Inhibitors: A Multicenter Retrospective Cohort Study in Turkey

**DOI:** 10.3390/curroncol33060340

**Published:** 2026-06-06

**Authors:** Kadriye Başkurt, Orhun Akdoğan, Yasemin Sağdıç Karateke, İlknur Deliktaş Onur, Galip Can Uyar, Enes Yeşilbaş, Ozan Yazıcı, Bülent Yıldız, Cengiz Karaçin, Ömür Berna Çakmak Öksüzoğlu, Osman Sütçüoğlu

**Affiliations:** 1 Department of Medical Oncology, Ankara Etlik City Hospital, 06170 Ankara, Turkey; 2 Department of Medical Oncology, Gazi University School of Medicine, 06500 Ankara, Turkey; 3 Department of Medical Oncology, Eskişehir Osmangazi University School of Medicine, 26040 Eskişehir, Turkeydrbulentyildiz@hotmail.com (B.Y.); 4 Department of Medical Oncology, Dr. Abdurrahman Yurtaslan Ankara Oncology Training and Research Hospital, 06200 Ankara, Turkey

**Keywords:** immune checkpoint inhibitors, 30-day mortality, real-world outcomes, ECOG performance status, immunotherapy

## Abstract

Short-term mortality following the last dose of immune checkpoint inhibitors (ICIs) is an increasingly recognized real-world outcome measure, yet its clinical predictors remain poorly characterized. This multicenter retrospective study included 458 consecutive patients with advanced melanoma, non-small cell lung cancer, or renal cell carcinoma who received ICIs at four tertiary centers in Turkey between 2018 and 2023. The primary endpoint was 30-day mortality after the final ICI dose. Among 458 patients, 71 (15.5%) died within 30 days. Multivariable logistic regression identified ECOG performance status ≥ 2, number of metastatic sites ≥ 3, and log-transformed C-reactive protein-to-albumin ratio (log-CAR) as independent predictors of 30-day mortality in Model 1 (AUC 0.954), while ECOG PS ≥ 2, brain metastasis, metastatic sites ≥ 3, and log-NLR were independent predictors in Model 2 (AUC 0.912). In the lung cancer subgroup, log-CAR and NLR remained independent predictors while ECOG PS did not. Patients who died within 30 days had significantly shorter progression-free survival (1.18 vs. 4.63 months) and overall survival (2.30 vs. 14.39 months) compared with survivors. These findings suggest that routine assessment of inflammatory and nutritional biomarkers alongside tumor burden parameters may help identify patients at high risk of early mortality and inform the timing of supportive care in ICI-treated populations.

## Simple Summary

Immunotherapy has improved outcomes for many cancer patients, but not all patients benefit equally. In some cases, treatment is continued very close to the end of life, when the likelihood of benefit is low. This study examined how often patients died within 30 days after their last dose of immunotherapy and which clinical factors were associated with this early mortality. We analyzed real-world data from multiple oncology centers and found that a significant proportion of patients died within this short time period. Poor performance status was the strongest factor linked to early death, while age, cancer type, and treatment characteristics were not clearly associated. These findings highlight the importance of careful patient selection and timely decision-making when continuing or stopping immunotherapy. Better identification of patients unlikely to benefit may help avoid unnecessary treatment and support earlier integration of palliative care.

## 1. Introduction

The use of immunotherapy treatment, particularly the subgroup known as immune checkpoint inhibitors (ICIs), has revolutionized the approach to various cancer subtypes over the past decades. Immune checkpoint inhibitors encompass anti-PD-1 inhibitors, anti-PD-L1 inhibitors, and anti-CTLA-4 inhibitors. Current response rates vary between 15% and 40%, depending on the tumor subtype and target expression [1,2,3]. Consequently, a substantial proportion of patients derive limited benefit, underscoring the importance of appropriate patient selection particularly in individuals with aggressive disease, poor performance status, or during the end-of-life period where the likelihood of meaningful response is low.

Short-term mortality following systemic anticancer therapy has traditionally been examined as a pragmatic indicator of treatment futility and patterns of care near the end of life. In recent years, 30-day mortality after the last ICI dose has similarly been explored as a real-world outcome measure, reflecting patient selection, disease trajectory, and the timing of treatment discontinuation rather than serving as a direct benchmark of care quality. Despite increasing clinical interest, robust real-world data on early mortality in ICI-treated patients remain limited. While randomized controlled trials provide detailed information on immune-related toxicities, real-world morbidity and mortality may exceed rates reported in trial populations [4,5]. The adverse effects of ICIs are driven by immune dysregulation and are distinct from those of cytotoxic agents, requiring immunosuppressive management and careful patient monitoring [6,7]. Given the high cost of these agents and the absence of reliable predictors of benefit in frail or comorbid populations, appropriate patient selection before treatment initiation carries both clinical and economic weight. This challenge is compounded by the fact that ICIs are perceived as less toxic than chemotherapy and are therefore more likely to be continued in patients approaching end of life, in whom meaningful benefit is unlikely, a tendency that may distort the interpretation of 30-day mortality as a quality benchmark when applied to immunotherapy [8].

Inflammatory and nutritional markers derived from routine blood tests have been evaluated as prognostic tools in ICI-treated patients. The neutrophil-to-lymphocyte ratio (NLR), which reflects the balance between innate immune activation and adaptive immune function, has been associated with worse overall survival and progression-free survival across multiple tumor types receiving ICIs [9]. The C-reactive protein-to-albumin ratio (CAR), which integrates systemic inflammation and nutritional reserve into a single index, has similarly shown consistent prognostic value in this setting [10]. Despite their established role in predicting survival outcomes, whether these markers are independently associated with early mortality following the last ICI dose has not been examined in a real-world multicenter cohort.

The objective of this multi-center retrospective study was to describe real-world 30-day mortality after the last dose of ICIs and to identify demographic and clinical characteristics associated with short-term mortality in patients treated for advanced melanoma, non-small cell lung cancer, and renal cell carcinoma.

## 2. Materials and Methods

### 2.1. Study Design and Patient Cohort

**Figure 1:**
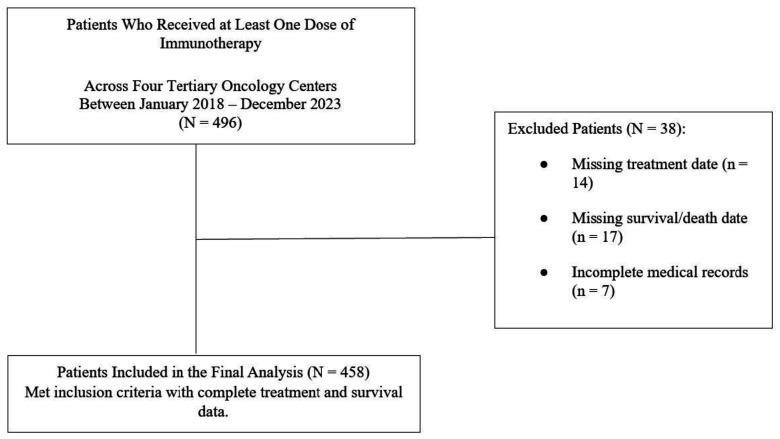
STROBE flow diagram of patient selection.

This multi-center retrospective study included all consecutive patients who received immune checkpoint inhibitors (ICIs) at four tertiary oncology centers in Turkey between 1 January 2018 and 30 December 2023. Eligible tumor types were metastatic melanoma, non–small cell lung cancer (NSCLC), and renal cell carcinoma (RCC). Patients participating in interventional clinical trials were excluded. A STROBE-compliant flow diagram (Figure 1) outlines the total number of screened patients, the number excluded due to missing treatment or survival data, and the final study cohort of 458 patients.

### 2.2. Data Collection and Variables

Data were extracted from electronic medical records and oncology clinic archives. Collected variables included demographic characteristics (age, sex, smoking status); cancer-related information (tumor type, stage, and sites of metastasis including brain and liver involvement, and number of metastatic sites); treatment characteristics (ICI agent, line of therapy (first-line vs. second-line and beyond), and treatment regimen (monotherapy vs. combination)); treatment intent (curative vs. palliative); and performance status based on the Eastern Cooperative Oncology Group (ECOG) scale. Comorbidity burden was quantified using the modified Charlson Comorbidity Index (mCCI), dichotomized as <8 vs. ≥8.

Baseline laboratory parameters collected within 30 days prior to ICI initiation included serum albumin (g/dL), C-reactive protein (CRP, mg/L), lactate dehydrogenase (LDH, U/L), and body mass index (BMI, kg/m^2^). Two composite inflammatory indices were derived from these values: the C-reactive protein-to-albumin ratio (CAR), calculated as CRP (mg/L) divided by albumin (g/dL), and the neutrophil-to-lymphocyte ratio (NLR), calculated as the absolute neutrophil count divided by the absolute lymphocyte count from the complete blood count obtained at the same time point.

Outcome data included the date of the last ICI administration, date of death, and documented cause of death. Patients receiving systemic treatment for stage IV disease were classified under palliative intent, while those treated without metastatic disease, including adjuvant or neoadjuvant settings, were categorized under curative intent.

### 2.3. Definition of 30-Day Mortality

The primary endpoint was 30-day mortality, defined as death occurring within 30 days after the final dose of immunotherapy, regardless of cancer type, treatment intent, or therapy line. Cause-of-death information was obtained from physician documentation; when unavailable, cases were classified as undetermined. Progression-free survival (PFS) and overall survival (OS) were both calculated from the date of ICI initiation to the date of disease progression or death from any cause, respectively, or censored at the last follow-up date.

### 2.4. Statistical Analysis

All statistical analyses were performed using IBM SPSS Statistics for Windows, version 25.0 (IBM Corp., Armonk, NY, USA) and Python version 3.14. Continuous variables were assessed for normality using the Shapiro–Wilk test and histogram inspection, expressed as medians with interquartile ranges (IQR), and compared between groups using the Mann–Whitney U or Kruskal–Wallis test. Categorical variables are presented as frequencies and percentages and compared using the Pearson chi-square or Fisher’s exact test.

Baseline CAR and NLR values were logarithmically transformed (log-CAR and log-NLR) prior to regression analyses due to their skewed distributions.

Univariate and multivariable logistic regression analyses were performed to identify predictors of 30-day mortality, with results reported as odds ratios (ORs) with 95% confidence intervals (CIs). Variables with *p* < 0.05 in univariate analysis were entered into the multivariable models. Given the collinearity between log-CAR and log-NLR, two separate multivariable models were constructed: Model 1 with log-CAR and Model 2 with log-NLR. Multicollinearity was assessed using the Variance Inflation Factor (VIF); LDH was excluded from multivariable modeling despite univariate significance, as its VIF exceeded 5.0 due to collinearity with both the number of metastatic sites and log-CAR.

PFS and OS were calculated from the date of ICI initiation and estimated using Kaplan–Meier curves, with group comparisons by the log-rank test. Univariate and multivariable Cox proportional hazards regression models were constructed for both endpoints, with log-CAR and log-NLR evaluated in separate models (Model A and Model B) to prevent multicollinearity. Model discrimination was assessed using Harrell’s C-index and the Akaike Information Criterion (AIC).

For the 30-day mortality logistic models, discriminative ability was assessed by the area under the ROC curve (AUC), with paired AUC comparisons performed using the DeLong test. Calibration was evaluated using the Hosmer–Lemeshow goodness-of-fit test and McFadden’s pseudo-R^2^.

In the lung cancer subgroup, given the limited number of events (*n* = 53), multivariable logistic regression was restricted to ECOG PS and each inflammatory biomarker separately to satisfy the events-per-variable rule. A two-sided *p*-value of <0.05 was considered statistically significant.

### 2.5. Ethical Considerations

The study was approved by the Ethics Committee for Scientific Research and Evaluation of Ankara Etlik City Hospital (Decision No: AEŞH-BADEK-2024-454) and by institutional review boards of all participating centers. Owing to the retrospective design, informed consent was waived, and all data were anonymized prior to analysis.

## 3. Results

### 3.1. Patient Characteristics

A total of 458 consecutive patients who received ICI therapy during the study period were included in the analysis, of whom 71 (15.5%) died within 30 days of their last ICI dose. The cohort was predominantly male (*n* = 359, 78.4%) and under 65 years of age (*n* = 257, 56.1%), with a median age of 63.0 years (IQR 55.0–70.0). The majority of patients had an ECOG performance status of 0–1 (*n* = 340, 74.2%), while 118 (25.8%) had an ECOG PS ≥ 2. Most patients received ICI as second-line therapy or beyond (*n* = 403, 88.0%), and treatment intent was palliative in 448 patients (97.8%). A modified Charlson Comorbidity Index ≥8 was recorded in 329 patients (71.8%). Brain metastases were present in 91 patients (19.9%), liver metastases in 179 (39.1%), and 103 patients (22.5%) had three or more metastatic sites. Lung cancer was the most common tumor type (*n* = 300, 65.5%), followed by renal cell carcinoma (*n* = 88, 19.2%) and malignant melanoma (*n* = 61, 13.3%). Nivolumab was the most frequently administered agent (*n* = 412, 90.0%), and ICI monotherapy was used in the vast majority of patients. Baseline demographic, clinical, and laboratory characteristics are summarized in Table 1.

Patients who died within 30 days had higher median CRP (75.20 vs. 12.70 mg/L), lower median albumin (3.11 vs. 4.00 g/dL), higher median LDH (421.00 vs. 245.00 U/L), higher median CAR (23.62 vs. 3.20), and higher median NLR (3.90 vs. 1.92) compared with those who survived beyond 30 days.

### 3.2. Survival Outcomes

The overall median PFS from ICI initiation was 4.20 months (95% CI 3.73–4.67). Patients who survived beyond 30 days had a median PFS of 4.63 months (95% CI 4.14–5.05), compared with 1.18 months (95% CI 0.61–1.61) in those who died within 30 days (log-rank *p* < 0.001). Progression events were recorded in 331 of 387 survivors and in all 71 patients who died within 30 days.

**Figure 2:**
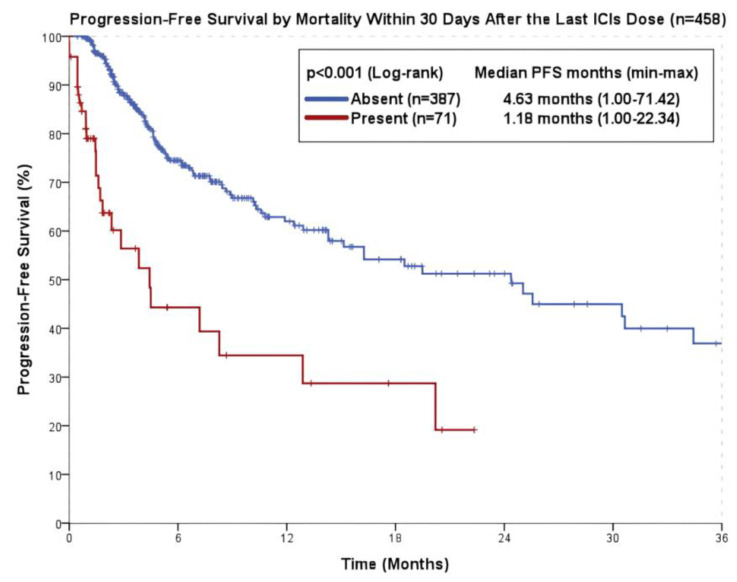
Kaplan-Meier Curve for Progression-Free Survival (PFS) Based on 30-Day Mortality Following the Last Dose of ICIs. ICIs: Immun Check Point Inhibitors, max: maximum, min: minimum *n*: sample size, PFS: Progression-Free Survival.

Overall survival from ICI initiation was also significantly shorter in the 30-day mortality group. The overall cohort median OS was 11.92 months (95% CI 10.11–13.74). Patients who survived beyond 30 days had a median OS of 14.39 months (95% CI 12.56–16.21), whereas those who died within 30 days had a median OS of 2.30 months (95% CI 1.81–2.78) (log-rank *p* < 0.001). Kaplan–Meier curves for PFS and OS stratified by 30-day mortality status are presented in Figure 2.

### 3.3. Predictors of 30-Day Mortality

In univariate logistic regression, ECOG PS ≥ 2 (OR 4.42, 95% CI 2.61–7.49, *p* < 0.001), brain metastasis (OR 5.75, 95% CI 3.33–9.91, *p* < 0.001), number of metastatic sites ≥ 3 (OR 4.24, 95% CI 2.48–7.22, *p* < 0.001), log-CAR (OR 9.71, 95% CI 2.98–27.25, *p* < 0.001), log-NLR (OR 2.09, 95% CI 1.30–5.35, *p* < 0.001), liver metastasis (OR 2.15, 95% CI 1.29–3.59, *p* = 0.003), LDH (OR 1.010, 95% CI 1.007–1.012, *p* < 0.001), BMI (OR 0.93, 95% CI 0.88–0.99, *p* = 0.023), and first-line ICI therapy (OR 0.43, 95% CI 0.22–0.83, *p* = 0.012) were significantly associated with 30-day mortality. Age, sex, smoking status, treatment intent, mCCI, diabetes mellitus, and coronary artery disease were not significant.

In multivariable Model 1 (incorporating log-CAR), ECOG PS ≥ 2 (OR 3.24, 95% CI 1.82–9.80, *p* = 0.033), number of metastatic sites ≥ 3 (OR 5.26, 95% CI 1.36–20.38, *p* = 0.016), and log-CAR (OR 7.29, 95% CI 2.44–23.12, *p* = 0.009) emerged as independent predictors of 30-day mortality. In multivariable Model 2 (incorporating log-NLR), ECOG PS ≥ 2 (OR 4.77, 95% CI 1.58–14.43, *p* = 0.006), brain metastasis (OR 8.85, 95% CI 2.53–30.94, *p* < 0.001), number of metastatic sites ≥ 3 (OR 9.49, 95% CI 2.55–35.29, *p* < 0.001), and log-NLR (OR 5.80, 95% CI 1.30–23.64, *p* = 0.023) were independent predictors. Univariate and multivariable analyses are presented in Table 2.

Model 1 demonstrated superior discriminative performance, with an AUC of 0.954 (95% CI 0.921–0.987) compared with 0.912 (95% CI 0.875–0.949) for Model 2 (DeLong test *p* = 0.038). Both models showed good calibration on the Hosmer–Lemeshow test (Model 1: *p* = 0.601; Model 2: *p* = 0.419) and McFadden’s pseudo-R^2^ values of 0.801 and 0.757, respectively. Model performance metrics are summarized in Supplementary Table S1.

### 3.4. Prognostic Factors for PFS and OS

In multivariable Cox regression for PFS, ECOG PS ≥ 2 (HR 2.48, 95% CI 1.97–3.12, *p* < 0.001), brain metastasis (HR 1.32, 95% CI 1.04–1.68, *p* = 0.024), and log-CAR (HR 1.23, 95% CI 1.10–1.37, *p* < 0.001) were independent predictors in Model A. For OS, ECOG PS ≥ 2 (HR 3.15, 95% CI 2.42–4.11, *p* < 0.001), brain metastasis (HR 1.72, 95% CI 1.33–2.22, *p* < 0.001), number of metastatic sites ≥ 3 (HR 1.45, 95% CI 1.13–1.87, *p* = 0.004), and log-CAR (HR 1.20, 95% CI 1.07–1.36, *p* = 0.003) remained significant. Model A showed better fit than Model B for both PFS (C-index 0.675 vs. 0.673; AIC 4044.46 vs. 4053.55) and OS (C-index 0.733 vs. 0.728; AIC 3159.22 vs. 3164.26). Full Cox regression results are presented in Table 3 and Table S2.

### 3.5. Causes of 30-Day Mortality and Subgroup Analysis by Cause of Death

Among the 71 patients who died within 30 days, the most frequent causes were sepsis/infection (*n* = 23, 32.4%), disease progression (*n* = 21, 29.6%), and thromboembolic events (*n* = 14, 19.7%). Myocardial infarction accounted for 5 deaths (7.0%) and immunotherapy-related toxicity for 2 (2.8%). The cause of death could not be determined in 6 patients (8.5%), all of whom died out of hospital. Causes of mortality are summarized in Table 4.

A subgroup analysis comparing PFS among the three leading causes of death revealed median PFS of 1.18 months in patients who died of sepsis, 1.61 months in those who died of disease progression, and 1.01 months in those who died of thromboembolic events. No statistically significant difference was observed among these groups (Kruskal–Wallis *p* = 0.436).

### 3.6. Lung Cancer Subgroup Analysis

Among the 300 lung cancer patients, 53 (17.7%) died within 30 days of the last ICI dose. In univariate analysis, ECOG PS ≥ 2 (OR 3.65, *p* < 0.001), brain metastasis (OR 4.91, *p* < 0.001), number of metastatic sites ≥ 3 (OR 4.21, *p* < 0.001), LDH (*p* < 0.001), and log-CAR (OR 8.11, *p* < 0.001) were significantly associated with 30-day mortality. In the multivariable model adjusted for ECOG PS, log-CAR (OR 8.45, 95% CI 2.86–14.24, *p* = 0.007) and NLR (OR 5.88, 95% CI 1.26–10.40, *p* = 0.019) remained independent predictors, while ECOG PS did not retain significance (*p* = 0.152). For PFS, ECOG PS ≥ 2 (HR 1.84, 95% CI 1.42–2.38, *p* < 0.001) and log-CAR (HR 1.19, 95% CI 1.05–1.35, *p* = 0.006) were independent predictors. For OS, ECOG PS ≥ 2 (HR 2.47, 95% CI 1.81–3.37, *p* < 0.001), brain metastasis (HR 1.69, 95% CI 1.25–2.28, *p* = 0.001), and log-CAR (HR 1.15, 95% CI 1.01–1.31, *p* = 0.048) were independent predictors. Subgroup results are detailed in Supplementary Tables S3 and S4.

## 4. Discussion

Thirty-day mortality following the last ICI dose reflects the intersection of disease biology, patient frailty, and clinical decision-making at the end of life. In this multicenter cohort of 458 patients, 15.5% died within 30 days of their final ICI administration. In addition to confirming the prognostic relevance of ECOG performance status, the sole independent predictor identified in earlier single-center analyses, this study demonstrates that systemic inflammatory indices and tumor burden parameters are independent predictors of 30-day mortality, with the logistic regression model incorporating log-CAR achieving an AUC of 0.954 [4].

The 30-day mortality rate in this cohort exceeds that reported in studies examining broader systemic anticancer therapy populations. Zdenkowski et al. reported a 12% rate among patients receiving palliative chemotherapy, and Gilbar et al. observed 5.6% in an Australian regional cancer center [11,12]. Among studies specifically examining 30-day mortality following the last dose of systemic anticancer therapy, Cetin et al. reported an overall rate of 7% across 1937 patients receiving intravenous anticancer therapy at a single Turkish center, with ECOG performance status and BMI emerging as independent predictors [13]. The higher rate observed in our cohort likely reflects the predominance of palliative-intent treatment, the high proportion of lung cancer patients, and country-specific reimbursement patterns that restrict ICI use to later lines of therapy. In Turkey, nivolumab, which accounted for 90% of administrations in this cohort, is reimbursed exclusively as second-line treatment for advanced NSCLC, RCC, and melanoma, which may concentrate treatment use in a more heavily pretreated and clinically fragile population. Kerekes et al., in a national cohort of 242,371 patients, reported that more than one in fourteen patients received immunotherapy at the end of life, with this proportion increasing over time across all cancer types [14].

ECOG performance status ≥ 2 was an independent predictor of 30-day mortality in both multivariable logistic regression models. This finding is consistent with prior evidence. Dall’Olio et al., in a meta-analysis of 19 studies comprising 3600 NSCLC patients, demonstrated that ECOG PS ≥ 2 was associated with significantly worse OS, PFS, and objective response rate in patients treated with ICIs (pooled OS HR 2.72, 95% CI 2.03–3.63) [15]. Mollica et al. similarly reported consistent survival detriment associated with poor performance status across multiple tumor types and ICI regimens in the MOUSEION-06 study [16]. However, in the lung cancer subgroup of the present study, ECOG PS did not retain independent significance once CAR and NLR were included in the multivariable model, suggesting that these objective laboratory indices may add prognostic value beyond performance status alone.

CAR was an independent predictor of 30-day mortality in both the overall cohort and the lung cancer subgroup, and Model 1, incorporating log-CAR, demonstrated superior discriminative performance compared with Model 2, incorporating log-NLR (AUC 0.954 vs. 0.912, DeLong test *p* = 0.038). CAR integrates CRP and albumin into a single index that reflects both systemic inflammatory burden and nutritional status, two biologically relevant determinants of treatment tolerance and disease trajectory. A meta-analysis by Dai and Wu, incorporating 11 studies and 1321 ICI-treated patients, reported that elevated CAR was independently associated with worse OS and PFS in ICI-treated patients [10]. The present study extends these findings by demonstrating that CAR is also independently associated with mortality within 30 days of the last ICI dose, a clinically distinct endpoint not previously examined in this context. NLR similarly emerged as an independent predictor across models. Xie et al., in a meta-analysis of 14 studies incorporating 1751 patients, reported that elevated pretreatment NLR was associated with worse OS and PFS across multiple tumor types receiving ICIs [9].

Tumor burden parameters, specifically brain metastasis and number of metastatic sites ≥ 3, were independent predictors of 30-day mortality in multivariable analysis. Brain metastasis is a well-recognized adverse prognostic factor in patients receiving ICIs, and its strong association with early mortality in the present study is consistent with the disproportionate representation of patients with intracranial disease among those receiving immunotherapy near the end of life [14]. The absence of a significant difference in PFS across the three leading causes of death suggests that patients who died within 30 days represented a uniformly high-risk population regardless of the terminal event, rather than distinct prognostic subgroups defined by cause of death.

Thromboembolic events represented a notable cause of early mortality in this cohort. The relationship between ICI therapy and venous thromboembolism has been examined in several studies, with Kartolo et al. and Sussman et al. reporting elevated thromboembolic event rates in ICI-treated patients [17,18]. Immune dysregulation induced by checkpoint inhibition may contribute to a prothrombotic state, and thromboembolic events may account for a proportion of deaths classified as undetermined in real-world cohorts.

The interpretation of 30-day mortality as a quality-of-care indicator requires caution when applied to immunotherapy. Roberts et al. demonstrated that patients who died within 30 days of systemic anticancer therapy were more likely to have received immunotherapy or targeted therapy as their final treatment line, and argued that differential interpretation is required when applying this benchmark to non-cytotoxic agents [8]. Unlike cytotoxic chemotherapy, ICIs are perceived as less toxic and may therefore be continued in patients approaching end of life even when the probability of meaningful benefit is low. In this cohort, the combination of poor performance status, high tumor burden, and elevated inflammatory indices identifies a patient profile in whom early transition to supportive care warrants consideration.

This study has several limitations. The retrospective design introduces selection bias and restricts the depth of available clinical data. Steroid use and antibiotic administration were not systematically recorded; both may independently affect inflammatory biomarker values and infectious mortality, and their absence is a confounding factor that prospective studies should address. Nivolumab accounted for 90% of administrations, reflecting Turkish reimbursement policy rather than clinical preference, which limits applicability to settings where a broader range of ICIs is available. The cohort covers three tumor types at four Turkish tertiary centers, and these findings require external validation before they can guide clinical decision-making more broadly.

## 5. Conclusions

In this multicenter real-world cohort, 15.5% of patients died within 30 days of their last ICI dose. Poor ECOG performance status, high tumor burden, and elevated CAR and NLR were independent predictors of early mortality. In the lung cancer subgroup, CAR and NLR remained independent predictors while ECOG PS did not, suggesting that objective laboratory indices may add prognostic value beyond performance status alone. Routine assessment of these inflammatory markers before ICI initiation may help identify patients at high risk of early mortality and inform the timing of supportive care.

## Figures and Tables

**Table 1:** Baseline Demographic, Clinical, and Laboratory Characteristics of Patients Stratified by 30-Day Mortality After the Last Dose of ICI Therapy.

Parameters	Overall (*N* = 458) (%)	30-Day Mortality Present (*n* = 71) (%)	30-Day Mortality Absent (*n* = 387) (%)
**Demographic Characteristics**			
**Age, Median (IQR)**	63.0 (55.0–70.0)	62.0 (52.5–68.5)	63.0 (55.5–70.0)
<65 years	257 (56.1)	38 (53.5)	219 (56.6)
≥65 years	201 (43.9)	33 (46.5)	168 (43.4)
**Gender**			
Male	359 (78.4)	58 (81.7)	301 (77.8)
Female	99 (21.6)	13 (18.3)	86 (22.2)
**Smoking Status**			
Never/Ex-smoker	104 (22.7)	14 (19.7)	90 (23.3)
Current smoker	354 (77.3)	57 (80.3)	297 (76.7)
**Clinical & Tumor Characteristics**			
**ECOG Performance Status**			
0–1	340 (74.2)	33 (46.5)	307 (79.3)
≥2	118 (25.8)	38 (53.5)	80 (20.7)
**Treatment Intention**			
Palliative intention	448 (97.8)	70 (98.6)	378 (97.7)
Curative intention	10 (2.2)	1 (1.4)	9 (2.3)
**Line of Immunotherapy (ICI)**			
First-line	55 (12.0)	15 (21.1)	40 (10.3)
Second-line and beyond	403 (88.0)	56 (78.9)	347 (89.7)
**Immunotherapy Type**			
Nivolumab	412 (90.0)	66 (93.0)	346 (89.4)
Pembrolizumab	38 (8.3)	4 (5.6)	34 (8.8)
Atezolizumab	6 (1.3)	1 (1.4)	5 (1.3)
Avelumab	1 (0.2)	0 (0.0)	1 (0.3)
Durvalumab	1 (0.2)	0 (0.0)	1 (0.3)
**Modified Charlson Comorbidity Index (mCCI)**			
<8	129 (28.2)	15 (21.1)	114 (29.5)
≥8	329 (71.8)	56 (78.9)	273 (70.5)
**Diabetes Mellitus (DM)**			
Present	86 (18.8)	15 (21.1)	71 (18.3)
Absent	372 (81.2)	56 (78.9)	316 (81.7)
**Coronary Artery Disease (CAD)**			
Present	69 (15.1)	15 (21.1)	54 (14.0)
Absent	389 (84.9)	56 (78.9)	333 (86.0)
**Cancer Type**			
Lung Cancer	300 (65.5)	53 (74.6)	247 (63.8)
Malignant Melanoma	61 (13.3)	6 (8.5)	55 (14.2)
Renal Cell Carcinoma	88 (19.2)	11 (15.5)	77 (19.9)
Urothelial Carcinoma	4 (0.9)	1 (1.4)	3 (0.8)
Gastrointestinal Cancer	3 (0.7)	0 (0.0)	3 (0.8)
Head and Neck Cancer	1 (0.2)	0 (0.0)	1 (0.3)
**Metastatic Tumor Burden**			
**Brain Metastasis**			
Absent	367 (80.1)	36 (50.7)	331 (85.5)
Present	91 (19.9)	35 (49.3)	56 (14.5)
**Liver Metastasis**			
Absent	279 (60.9)	32 (45.1)	247 (63.8)
Present	179 (39.1)	39 (54.9)	140 (36.2)
**Number of Metastatic Sites**			
<3 sites	355 (77.5)	37 (52.1)	318 (82.2)
≥3 sites	103 (22.5)	34 (47.9)	69 (17.8)
**Baseline Laboratory Parameters**			
**BMI (kg/m^2^), Median (IQR)**	24.80 (22.09–26.76)	22.86 (20.84–25.40)	25.14 (22.13–27.06)
**Albumin (g/dL), Median (IQR)**	3.93 (3.62–4.22)	3.11 (2.87–3.41)	4.00 (3.77–4.26)
**CRP (mg/L), Median (IQR)**	14.50 (8.62–25.85)	75.20 (50.20–99.20)	12.70 (7.75–19.20)
**LDH (U/L), Median (IQR)**	256.00 (197.00–321.00)	421.00 (277.50–612.00)	245.00 (190.50–299.00)
**CAR, Median (IQR)**	3.71 (2.14–6.63)	23.62 (15.28–33.12)	3.20 (1.94–4.67)
**NLR, Median (IQR)**	2.14 (1.59–2.91)	3.90 (3.14–5.92)	1.92 (1.52–2.48)

Abbreviations: BMI, Body Mass Index; CAR, C-Reactive Protein-to-Albumin Ratio; CRP, C-Reactive Protein; ECOG, Eastern Cooperative Oncology Group; IQR, Interquartile Range; LDH, Lactate Dehydrogenase; NLR, Neutrophil-to-Lymphocyte Ratio.

**Table 2:** Univariate and Multivariate Logistic Regression Analyses for Factors Associated with 30-Day Mortality After the Last Dose of Immune Checkpoint Inhibitors.

Variables	Univariate Analysis OR (95% CI)	*p*-Value	Multivariate Model 1 (log-CAR) OR (95% CI)	*p*-Value	Multivariate Model 2 (NLR) OR (95% CI)	*p*-Value
Age (≥65 vs. <65 years)	1.13 (0.68–1.88)	0.632	—	—	—	—
Gender (Male vs. Female)	1.27 (0.67–2.44)	0.462	—	—	—	—
Smoking Status (Current vs. Never/Ex)	1.23 (0.66–2.32)	0.514	—	—	—	—
ECOG PS (≥2 vs. 0–1)	4.42 (2.61–7.49)	<0.001	3.24 (1.82–9.80)	0. 033	4.77 (1.58–14.43)	0.006
Treatment Intention (Palliative vs. Curative)	1.67 (0.21–13.36)	0.631	—	—	—	—
Line of ICI (≥2nd vs. 1st line)	0.43 (0.22–0.83)	0.012	0.51 (0.12–2.19)	0.367	0.52 (0.06–1.86)	0.070
MCCI (≥8 vs. <8)	1.56 (0.85–2.87)	0.154	—	—	—	—
Diabetes Mellitus (Yes vs. No)	1.19 (0.64–2.23)	0.582	—	—	—	—
Coronary Artery Disease (Yes vs. No)	1.65 (0.87–3.13)	0.123	—	—	—	—
Brain Metastasis (Present vs. Absent)	5.75 (3.33–9.91)	<0.001	3.67 (0.79–16.98)	0.096	8.85 (2.53–30.94)	<0.001
Liver Metastasis (Present vs. Absent)	2.15 (1.29–3.59)	0.003	2.42 (0.71–8.22)	0.155	2.12 (0.68–6.55)	0.193
Number of Metastatic Sites (≥3 vs. <3)	4.24 (2.48–7.22)	<0.001	5.26 (1.36–20.38)	0.016	9.49 (2.55–35.29)	<0.001
BMI (per 1 kg/m^2^ increase)	0.93 (0.88–0.99)	0.023	0.90 (0.79–1.04)	0.149	0.89 (0.78–1.01)	0.075
LDH (per unit increase)	1.010 (1.007–1.012)	<0.001	—	—	—	—
log-CAR (per unit increase)	9.71 (2.98–27.25)	<0.001	7.29 (2.44–23.12)	0.009	—	—
log-NLR (per unit increase)	2.09 (1.30–5.35)	<0.001	—	—	5.80 (1.30–23.64)	0.023

Abbreviations: BMI, Body Mass Index; CAR, C-Reactive Protein-to-Albumin Ratio; CI, Confidence Interval; ECOG PS, Eastern Cooperative Oncology Group Performance Status; ICI, Immune Checkpoint Inhibitor; MCCI, Modified Charlson Comorbidity Index; NLR, Neutrophil-to-Lymphocyte Ratio; OR, Odds Ratio. Note 1: Variables with *p* < 0.05 in univariate analysis were included in the multivariable logistic regression models. Due to collinearity between CAR and NLR, separate multivariate models were constructed. Note 2: Multicollinearity among independent variables included in the final multivariable models was evaluated using the Variance Inflation Factor (VIF). All variables retained in the multivariable models exhibited VIF values < 2 (ranging from 1.01 to 1.11), indicating no significant multicollinearity. Baseline LDH, although highly significant in univariate analysis (*p* < 0.001), exhibited a VIF > 5.0 due to critical multicollinearity with both the number of metastatic sites (tumor burden) and log-CAR (systemic inflammation). Consequently, to prevent variance inflation and preserve model stability, LDH was excluded from the multivariable steps.

**Table 3:** Multivariate Cox Proportional Hazards Regression Analyses for Progression-Free Survival (PFS) and Overall Survival (OS) from ICI Initiation.

Predictor Variables	Model A (log-CAR) Hazard Ratio (95% CI)	Model A (log-CAR) *p*-Value	Model B (log-NLR) Hazard Ratio (95% CI)	Model B (log-NLR) *p*-Value
**Progression-Free Survival (PFS)**				
Age (≥65 vs. <65 years)	1.16 (0.95–1.42)	0.142	1.13 (0.92–1.38)	0.255
ECOG PS (≥2 vs. 0–1)	2.48 (1.97–3.12)	<0.001	2.53 (2.01–3.19)	<0.001
Brain Metastasis (Present vs. Absent)	1.32 (1.04–1.68)	0.024	1.34 (1.05–1.71)	0.018
Number of Metastatic Sites (≥3 vs. <3)	1.23 (0.97–1.56)	0.095	1.27 (1.00–1.61)	0.049
BMI (per 1 kg/m^2^ increase)	0.98 (0.96–1.00)	0.109	0.98 (0.96–1.00)	0.061
log-CAR (per unit increase)	1.23 (1.10–1.37)	<0.001	—	—
log-NLR (per unit increase)	—	—	1.31 (1.03–1.67)	0.028
**Overall Survival (OS)**				
Age (≥65 vs. <65 years)	1.22 (0.97–1.53)	0.091	1.21 (0.96–1.52)	0.106
ECOG PS (≥2 vs. 0–1)	3.15 (2.42–4.11)	<0.001	3.17 (2.43–4.12)	<0.001
Brain Metastasis (Present vs. Absent)	1.72 (1.33–2.22)	<0.001	1.78 (1.38–2.29)	<0.001
Number of Metastatic Sites (≥3 vs. <3)	1.45 (1.13–1.87)	0.004	1.55 (1.21–1.98)	<0.001
log-CAR (per unit increase)	1.20 (1.07–1.36)	0.003	—	—
log-NLR (per unit increase)	—	—	1.33 (1.01–1.77)	0.043

Abbreviations: BMI, Body Mass Index; CAR, C-Reactive Protein-to-Albumin Ratio; CI, Confidence Interval; ECOG PS, Eastern Cooperative Oncology Group Performance Status; HR, Hazard Ratio; ICI, Immune Checkpoint Inhibitor; NLR, Neutrophil-to-Lymphocyte Ratio; OS, Overall Survival; PFS, Progression-Free Survival. Note: Due to collinearity, log-CAR and log-NLR were evaluated in separate multivariate Cox regression models. Covariates that achieved statistical significance (*p* < 0.05) in the univariate analyses were included in the respective multivariate models. Multicollinearity among independent variables included in the final multivariable Cox proportional hazards models was evaluated using the Variance Inflation Factor (VIF). All variables retained in the multivariable models exhibited VIF values < 2 (ranging from 1.01 to 1.11), indicating no significant multicollinearity. Baseline LDH, although highly significant in univariate analysis (*p* < 0.001), exhibited a VIF > 5.0 due to critical multicollinearity with both the number of metastatic sites (tumor burden) and log-CAR (systemic inflammation). Consequently, to prevent variance inflation and preserve model stability, LDH was excluded from the multivariable Cox regression steps. Model Performance and Comparison: The predictive accuracy and goodness-of-fit of the multivariate Cox regression models were evaluated using Harrell’s C-index and the Akaike Information Criterion (AIC), respectively. For Overall Survival (OS), Model A (incorporating log-CAR) demonstrated strong discrimination and superior model fit (C-index: 0.733, AIC: 3159.22) compared to Model B (incorporating log-NLR) (C-index: 0.728, AIC: 3164.26). Similarly, for Progression-Free Survival (PFS), Model A exhibited better predictive performance (C-index: 0.675, AIC: 4044.46) than Model B (C-index: 0.673, AIC: 4053.55).

**Table 4:** Causes of 30-Day Mortality After the Last Dose of ICI Therapy.

Causes of Mortality	*n* = 71	%
Sepsis/Infection	23	32.4
Disease Progression	21	29.6
Thromboembolic event	14	19.7
Unknown/Not available	6	8.5
Myocardial infarction	5	7.0
Immunotherapy-related toxicity	2	2.8

Note: The specific cause of mortality for 6 patients could not be definitively determined as these events occurred out-of-hospital (at home).

## Data Availability

The datasets generated and/or analyzed during the current study are not publicly available due to patient privacy and ethical considerations but are available from the corresponding author on reasonable request.

## Supplementary Materials

The following supporting information can be downloaded at: https://www.mdpi.com/article/10.3390/curroncol33060340/s1, Table S1: Predictive Performance, Calibration, and Comparison of the Multivariate Models for 30-Day Mortality After the Last Dose of ICI Therapy. Table S2: Univariate Cox Proportional Hazards Regression Analyses for Progression-Free Survival (PFS) and Overall Survival (OS) from ICI Initiation. Table S3: Subgroup Analysis of Lung Cancer Patients (*n* = 300): Univariate and Multivariate Logistic Regression for 30-Day Mortality. Table S4: Subgroup Analysis of Lung Cancer Patients (*n* = 300): Cox Proportional Hazards Regression for PFS and OS.
