# Supplementary material for: Real-World 30-Day Mortality After the Last Dose of Immune Checkpoint Inhibitors: A Multicenter Retrospective Cohort Study in Turkey
Source: Curr Oncol. 2026 Jun 6;33(6):340. doi: 10.3390/curroncol33060340 (PMC13298277; doi:10.3390/curroncol33060340)
Supplement: Supplementary file 1 [file curroncol-33-00340-s001.zip › curroncol-4353218-supplementary.pdf]

Supplementary Table S1. Predictive Performance, Calibration, and Comparison of the Multivariate Models for 30-Day Mortality After the Last Dose of ICI Therapy.

Performance Metrics	Model 1 (with log-CAR)	Model 2 (with log-NLR)	p-value (Between Models) *
**Discrimination**			
**Area Under the Curve (AUC)**	0.954 (0.921–0.987)	0.912 (0.875–0.949)	**0.038**
**Sensitivity (%)**	92.5	88.3	—
**Specificity (%)**	89.1	85.6	—
**Model Fit & Calibration**			
**Pseudo R^2 (McFadden)**	0.801	0.757	—
**Akaike Information Criterion (AIC)**	92.73	109.87	—
**Hosmer-Lemeshow Goodness-of-Fit**	$X^2 = 6.42$, p = 0.601	$X^2 = 8.15$, p = 0.419	—

* Comparison of AUCs between Model 1 and Model 2 was performed using the DeLong test.

Note 1: Lower AIC values indicate a better model fit. Model 1 demonstrated a superior fit compared to Model 2. Note 2: For the Hosmer-Lemeshow test, a p-value> 0.05 indicates good calibration (no significant difference between predicted and observed outcomes).

Supplementary Table S2. Univariate Cox Proportional Hazards Regression Analyses for Progression-Free Survival (PFS) and Overall Survival (OS) from ICI Initiation.

Predictor Variables	PFS Hazard Ratio (95% CI)	PFS p-value	OS Hazard Ratio (95% CI)	OS p-value
**Age (≥65 vs. <65 years)**	**1.27 (1.04–1.55)**	**0.018**	**1.38 (1.10–1.72)**	**0.005**
Gender (Male vs. Female)	1.18 (0.93–1.50)	0.180	1.15 (0.88–1.50)	0.315
**ECOG PS (≥2 vs. 0–1)**	**2.73 (2.19–3.42)**	**<0.001**	**3.81 (2.95–4.91)**	**<0.001**
Line of ICI (≥2nd vs. 1st line)	0.84 (0.63–1.12)	0.230	0.73 (0.53–1.00)	0.183
**Brain Metastasis (Present vs. Absent)**	**1.43 (1.13–1.81)**	**0.003**	**2.12 (1.65–2.71)**	**<0.001**
Liver Metastasis (Present vs. Absent)	1.12 (0.92–1.37)	0.266	1.24 (1.00–1.55)	0.105
**Number of Metastatic Sites (≥3 vs. <3)**	**1.46 (1.16–1.83)**	**0.001**	**1.94 (1.53–2.46)**	**<0.001**
**BMI (per 1 kg/m^2 increase)**	**0.98 (0.95–1.00)**	**0.035**	0.99 (0.96–1.01)	0.250
LDH (per unit increase)	1.008 (1.003–1.013)	**<0.001**	1.011 (1.004–1.018)	**<0.001**
**log-CAR (per unit increase)**	**1.35 (1.21–1.51)**	**<0.001**	**1.46 (1.29–1.65)**	**<0.001**
**log-NLR (per unit increase)**	**1.62 (1.27–2.05)**	**<0.001**	**2.02 (1.54–2.65)**	**<0.001**

Abbreviations: BMI, Body Mass Index; CAR, C-Reactive Protein-to-Albumin Ratio; CI, Confidence Interval; ECOG PS, Eastern Cooperative Oncology Group Performance Status; HR, Hazard Ratio; ICI, Immune Checkpoint Inhibitor; NLR, Neutrophil-to-Lymphocyte Ratio; OS, Overall Survival; PFS, Progression-Free Survival.

Supplementary Table S3. Subgroup Analysis of Lung Cancer Patients (*n* = 300): Univariate and Multivariate Logistic Regression for 30-Day Mortality.

Predictor Variables	Univariate OR (95% CI)	p-value	Multivariate OR (95% CI)	p-value
Age (≥65 vs. <65 years)	1.04 (0.57–1.88)	0.908	—	—
Gender (Male vs. Female)	1.15 (0.51–2.62)	0.735	—	—
ECOG PS (≥2 vs. 0–1)	3.65 (1.97–6.75)	<0.001	1.09 (0.40–2.58)	0.152
Line of ICI (≥2nd vs. 1st line)	1.69 (0.63–4.53)	0.296	—	—
Brain Metastasis (Present vs. Absent)	4.91 (2.59–9.32)	<0.001	—	—
Liver Metastasis (Present vs. Absent)	1.49 (0.82–2.70)	0.190	—	—
**Number of Metastatic Sites (≥3 vs. <3)**	**4.21 (2.25–7.87)**	**<0.001**	**1.89 (0.43–3.34)**	**0.011**
BMI (per 1 kg/m^2 increase)	0.94 (0.87–1.00)	0.067	0.97 (0.97–1.02)	0.234
LDH (per unit increase)	1.007 (1.002–1.012)	**<0.001**	—	—
**Log-CAR (per unit increase)**	**8.11 (3.58–12.38)**	**<0.001**	**8.45 (2.86–14.24)**	**0.007**
**NLR (per unit increase) ***	**3.65 (1.31–8.09)**	**<0.001**	**5.88 (1.26–10.40)**	**0.019**

Abbreviations: BMI, Body Mass Index; CAR, C-Reactive Protein-to-Albumin Ratio; CI, Confidence Interval; ECOG PS, Eastern Cooperative Oncology Group Performance Status; ICI, Immune Checkpoint Inhibitor; NLR, Neutrophil-to-Lymphocyte Ratio; OR, Odds Ratio.

Note 1: To prevent multicollinearity, log-CAR and NLR were evaluated in separate multivariate models. For 30-day mortality, due to the limited number of events (n=53) in the lung cancer subgroup, the multivariable logistic regression models were parsimoniously adjusted only for the most robust clinical confounder (ECOG PS) to satisfy the 'events-per-variable' rule and prevent model overfitting. Both biomarkers remained strongly independent predictors of early mortality.

Note 2: Raw NLR was utilized in the logistic regression model instead of log-NLR to prevent perfect prediction (quasi-complete separation) phenomena. Multicollinearity among independent variables included in the final multivariable models was evaluated using the Variance Inflation Factor (VIF). All variables retained in the multivariable models exhibited VIF values <2.0 (ranging from 1.01 to 1.11), indicating no significant multicollinearity. Baseline LDH, although highly significant in univariate analysis (p< 0.001), exhibited a VIF>5.0 due to critical multicollinearity with both the number of metastatic sites (representing tumor burden) and log-CAR (representing systemic inflammation). Consequently, to prevent variance inflation and preserve model stability, LDH was excluded from the multivariable logistic regression steps.

Supplementary Table S4. Subgroup Analysis of Lung Cancer Patients (*n* = 300): Cox Proportional Hazards Regression for PFS and OS.

Predictor Variables	Univariate HR (95% CI)	p-value	Multivariate HR (95% CI) *	p-value
**Progression-Free Survival (PFS)**				
Age (≥65 vs. <65 years)	1.19 (0.94–1.50)	0.154	—	—
Gender (Male vs. Female)	0.95 (0.69–1.30)	0.737	—	—
**ECOG PS (≥2 vs. 0–1)**	**1.95 (1.51–2.53)**	**<0.001**	**1.84 (1.42–2.38)**	**<0.001**
Line of ICI (≥2nd vs. 1st line)	1.49 (1.06–2.09)	0.021	1.33 (0.94–1.88)	0.101
Brain Metastasis (Present vs. Absent)	1.25 (0.94–1.66)	0.117	—	—
Number of Metastatic Sites (≥3 vs. <3)	1.20 (0.92–1.57)	0.184	—	—
LDH (per unit increase)	1.004 (1.001–1.007)	**<0.001**	—	—
**log-CAR (per unit increase)**	**1.23 (1.08–1.39)**	**0.001**	**1.19 (1.05–1.35)**	**0.006**
log-NLR (per unit increase)	1.29 (0.99–1.68)	0.062	1.21 (0.93–1.57)	0.149
**Overall Survival (OS)**				
Age (≥65 vs. <65 years)	1.21 (0.94–1.57)	0.140	—	—
Gender (Male vs. Female)	0.81 (0.57–1.14)	0.221	—	—
**ECOG PS (≥2 vs. 0–1)**	**2.77 (2.08–3.69)**	**<0.001**	**2.47 (1.81–3.37)**	**<0.001**
Line of ICI (≥2nd vs. 1st line)	1.59 (1.08–2.32)	0.017	1.46 (0.99–2.15)	0.055
**Brain Metastasis (Present vs. Absent)**	**1.80 (1.34–2.41)**	**<0.001**	**1.69 (1.25–2.28)**	**0.001**
Number of Metastatic Sites (≥3 vs. <3)	1.49 (1.13–1.98)	0.005	1.26 (0.94–1.69)	0.125
LDH (per unit increase)	1.009 (1.006–1.012)	**<0.001**	—	—
**log-CAR (per unit increase)**	**1.29 (1.13–1.48)**	**<0.001**	**1.15 (1.01–1.31)**	**0.048**
log-NLR (per unit increase)	1.48 (1.09–2.01)	0.012	1.05 (0.78–1.41)	0.744

Abbreviations: CAR, C-Reactive Protein-to-Albumin Ratio; CI, Confidence Interval; ECOG PS, Eastern Cooperative Oncology Group Performance Status; HR, Hazard Ratio; ICI, Immune Checkpoint Inhibitor; NLR, Neutrophil-to-Lymphocyte Ratio; OS, Overall Survival; PFS, Progression-Free Survival.

* Note: To prevent multicollinearity, log-CAR and log-NLR were evaluated in separate multivariate Cox regression models. The values in the multivariate column for the clinical covariates (ECOG, Line of ICI, Brain Metastasis, etc.) are derived from the primary log-CAR model. Both log-CAR and log-NLR values reflect their independent prognostic power when adjusted for these respective clinical variables. Multicollinearity among independent variables included in the final multivariable models was evaluated using the Variance Inflation Factor (VIF). All variables retained in the multivariable models exhibited VIF values <2.0 (ranging from 1.01 to 1.11), indicating no significant multicollinearity. Baseline LDH, although highly significant in univariate analysis (p< 0.001), exhibited a VIF>5.0 due to critical multicollinearity with both the number of metastatic sites (representing tumor burden) and log-CAR (representing systemic inflammation). Consequently, to prevent variance inflation and preserve model stability, LDH was excluded from the multivariable logistic regression steps.
